# Pediatric endoscopic retrograde pancreatography expertise in chronic pancreatitis: a single-center analysis

**DOI:** 10.3389/fped.2025.1491579

**Published:** 2025-02-11

**Authors:** Hongxi Guo, Juan Luo, Hu Yang, Jun Yang, Hongqiang Bian, Xufei Duan, Xin Wang

**Affiliations:** ^1^ Department of General Surgery, Wuhan Children’s Hospital, Tongji Medical College, Huazhong University of Science & Technology, Wuhan, China; ^2^ Department of Endocrinology and Metabolism, Wuhan Children’s Hospital, Tongji Medical College, Huazhong University of Science & Technology, Wuhan, China

**Keywords:** endoscopic retrograde pancreatography (ERP), children, chronic pancreatitis (CP), pancreas divisum, pseudocyst

## Abstract

**Background and aim:**

Chronic pancreatitis (CP) in children has exhibited an annual increase in incidence in recent years. Pediatric CP presents unique clinical features compared to adult cases. Endoscopic retrograde pancreatography (ERP) serves as a valuable and safe tool for diagnosing and treating CP in adults. However, data on endoscopic treatment of CP in children are still limited.

**Methods:**

Demographics, etiology, surgical indications, diagnosis, treatment details, associated complications, and follow-up information were retrospectively studied in consecutive patients (<18 years old) who underwent ERP for CP between January 2020 and October 2024.

**Results:**

A total of 17 children (7 male, 10 female) with a mean age of 10.0 ± 2.7 years were included in the study. A total of 34 endoscopic treatments were conducted. Recurrent abdominal pain was the primary clinical symptom. Imaging predominantly revealed pancreatic duct abnormalities such as tortuous dilatation and the presence of pancreatic duct stones. Notably, 41.2% (7 cases) involved genetic and congenital anatomical variations. Pancreatic duct stent placement was successfully performed in all 17 children (100.0% success rate). Stent replacements occurred on average 2.2 times (range 1–5) at intervals of 3–6 months. Postoperative pancreatitis developed in 2 cases (5.9%, 2/34), and hyperamylasemia occurred in 5 cases (14.7%, 5/34). The postprocedure visual analogue scale (VAS) score for abdominal pain significantly decreased from 6 to 1 (*P* < 0.001). The annual frequency of pancreatitis episodes showed a significant reduction, decreasing from 2.4 times pre-treatment to 0.6 times post-treatment (*P* < 0.05). Body mass index (BMI) also showed a significant improvement post-treatment compared to pre-treatment (*P* < 0.05).

**Conclusions:**

ERP performed by trained endoscopists utilizing standard adult endoscopes and accessories proved a safe and effective treatment option for pediatric CP, with complication rates comparable to those reported in adult cases.

## 1 Introduction

Chronic pancreatitis (CP) is a distinct pathological entity, not merely a progression from acute pancreatitis. It represents a fibroinflammatory condition of the pancreas with a multifactorial etiology. Historically, diagnosing CP in the pediatric population has been uncommon. Untreated CP can lead to irreversible structural changes within the pancreas, manifesting as pain syndromes (both recurrent and persistent). Furthermore, it can progress to endocrine and exocrine insufficiency (1). In recent years, CP has received growing clinical attention due to its significant impact on quality of life. Recurrent abdominal pain, malnutrition, and frequent hospitalizations associated with CP impose a substantial burden on both patients and their families, as well as on society (2, 3). Reported etiologies of CP encompass hereditary factors, anatomical anomalies, exposure to toxins or medications, metabolic disturbances, and autoimmunity. Notably, up to 20% of patients may have a combination of these risk factors (4). Genetic mutations, including those in *PRSS1*, *SPINK1*, *CFTR*, and *CTRC* genes, and anatomical anomalies such as pancreas divisum (PD), pancreaticobiliary maljunction (PBM), and sphincter dysfunction, are the most prevalent risk factors for pediatric CP, with pancreas divisum being the most common (4). Symptoms of pediatric CP often present in a nonspecific manner and may have an insidious onset. Clinical features can vary depending on the disease stage. Common symptoms include abdominal pain, nausea, vomiting, and malnutrition, which can pose a significant challenge for early diagnosis. A step-wise treatment approach is increasingly becoming the standard for CP management. This approach begins with medical therapy, encompassing fasting, fluid resuscitation, acid suppression, enzyme replacement, and anti-infective therapy. If medical treatment proves unsuccessful, endoscopic interventions like pancreatic sphincterotomy and stent placement are considered. Finally, surgery becomes the next option if endoscopic treatment fails or is not feasible (1, 5). Endoscopic treatment for CP has demonstrated efficacy in adult populations (6). Emerging evidence suggests similarly favorable outcomes in children (7, 8). Endoscopic retrograde pancreatography (ERP) is a complex procedure with inherent complication rates. These challenges are further magnified in pediatric patients due to their unique physiology. Compared to adults, children have lower tolerance for the procedure, limited cooperation, narrower and more delicate gastrointestinal tracts, incompletely developed organs, and a decreased ability to withstand postprocedure complications. Additionally, pediatric CP often presents with earlier onset, prominent pancreatic duct calcification, and the presence of pancreatic stones and strictures. These factors are further compounded by the larger diameter of standard adult duodenoscopes used in ERP.

Therefore, careful patient selection is paramount for successful pediatric therapeutic ERP. Currently, the management of pediatric CP with ERP relies heavily on data extrapolated from adult studies. The paucity of safety data specific to the pediatric population remains a significant concern, particularly in studies from specialist pediatric hospitals.

This study retrospectively analyzes the experience of a pediatric specialist hospital in performing 34 ERP procedures on 17 children diagnosed with CP between June 2020 and June 2024. The study focuses on patient clinical presentation, diagnostic workup, treatment outcomes achieved through ERP, and associated safety considerations.

## 2 Methods and materials

### 2.1 Patients and setting

This retrospective study was performed at the Wuhan Children's Hospital, a national referral center for specialized pediatric endoscopic procedures within the Department of Pediatric General Surgery. ERCP (Endoscopic Retrograde Cholangiopancreatography) and ERP was introduced to our hospital in June 2020, and its utilization has since grown substantially in both frequency and complexity. Notably, our institution is among the limited number of centers in China offering ERP to the pediatric population.

Clinical data were retrospectively collected from August 2020 to June 2024. The study included children under 18 years of age diagnosed with CP who underwent ERP in the General Surgery Department of Wuhan Children's Hospital. The diagnosis of CP was established based on the criteria set forth by the International Study Group of Pediatric Pancreatitis: In Search for a Cure (INSPPIRE) (9). These criteria encompass histological features such as acinar and ductal tissue loss, periductal chronic inflammatory infiltration, periductal fibrosis, ductal obstruction, perineural inflammation, and relative paucity of islet cells. In some cases, the diagnosis was based on imaging findings of CP (pancreatic duct stones, multiple calcifications throughout the pancreas, irregular dilatation of the main pancreatic duct and scattered branch pancreatic duct dilations or obstructions due to stones or protein plugs, and dilatation of the main and branch pancreatic ducts) (10), combined with clinical manifestations of pancreatic-type abdominal pain and endocrine/exocrine pancreatic insufficiency. The primary indications for ERP in this study included the presence of pancreatic duct stones, strictures, pseudocysts, and pancreas divisum. These interventions aimed to alleviate pancreatic pain and improve patients' quality of life. Written informed consent for the ERP procedure was obtained from the legal guardians of all participating children. The study protocol received approval from the local ethics committee (reference number: 2024R032-E01).

### 2.2 Procedure and equipment

In preparation for ERP procedure, patients underwent a six-hour fast and received intravenous fluid therapy. One hour before the procedure, diclofenac sodium was routinely administered for the prevention of post-ERCP pancreatitis (PEP), at a dose of 1 mg/kg, with a maximum dose of 12.5 mg. A comprehensive preoperative evaluation was performed, including complete blood count, biochemistry panel, coagulation studies, abdominal ultrasound, and either CT or magnetic resonance cholangiopancreatography (MRCP), to determine the cause of CP and assess for pancreatic duct strictures or stones. Detailed preoperative communication with the child's family was conducted, and informed consent was obtained. Standard adult ERP equipment was utilized, including duodenoscopes (Olympus models JF-260V or TJF-260V), contrast catheters, guidewires, cutting balloons, stone retrieval baskets, pancreatic duct stents, and stone retrieval balloons (manufactured by Cook Medical and Anri).

Due to the challenges posed by limited patient cooperation, tolerance, and the delicate pediatric gastrointestinal tract, general anesthesia with endotracheal intubation was employed for all procedures. For surgeries exceeding two hours based on preoperative evaluation, urinary catheterization was implemented. Two experienced anesthesiologists ensured proper anesthesia delivery, airway management, and close monitoring of vital signs and end-tidal CO_2_ to detect and address any potential respiratory depression or apnea. Radiation protection safeguards were utilized for the thyroid and reproductive organs. Consistent communication throughout the operation between the endoscopist and anesthesiologists allowed for adjustments in anesthesia depth to optimize patient care and minimize postoperative recovery time.

**Figure 1:**
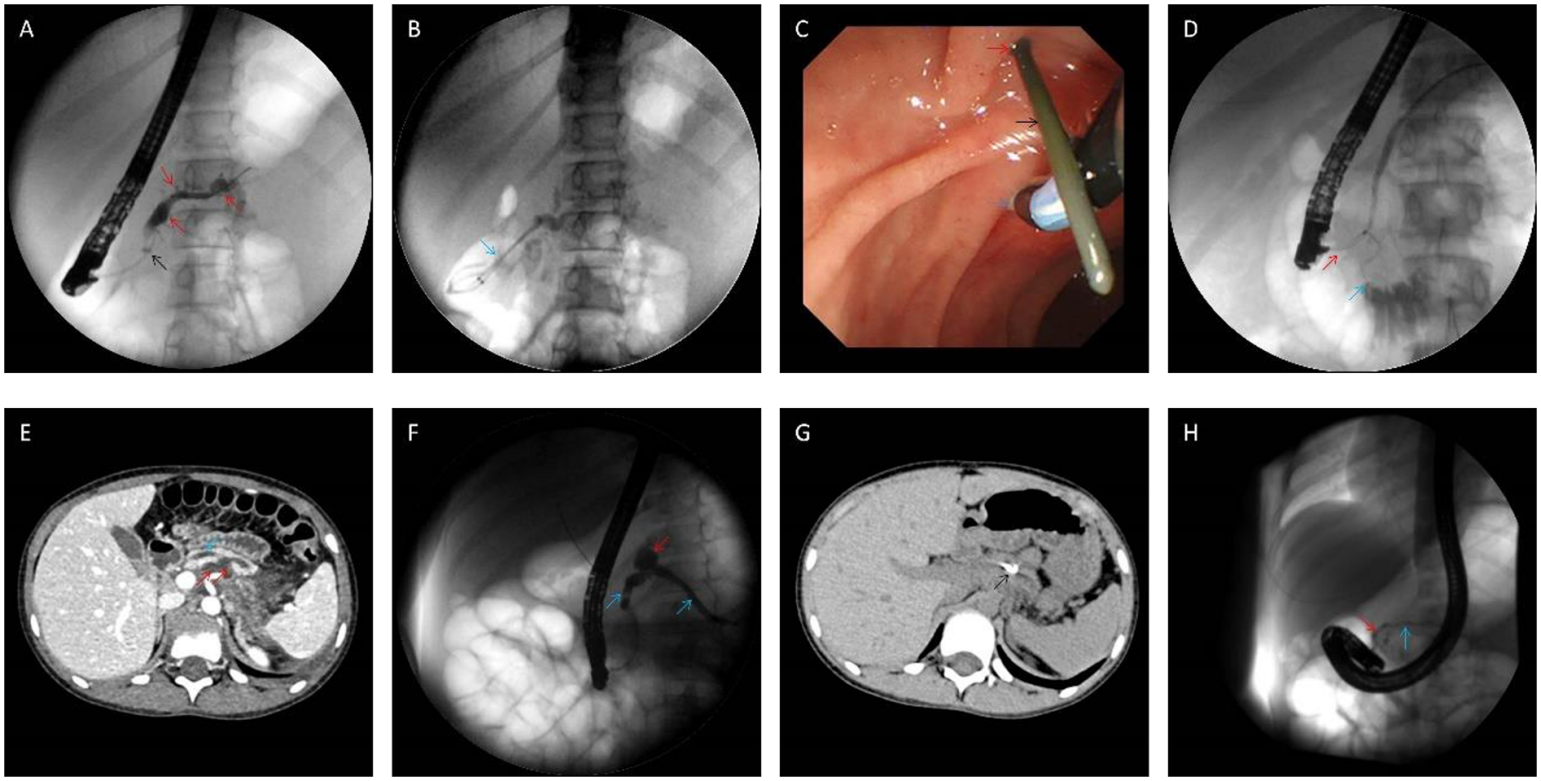
Intraoperative findings during endoscopic retrograde cholangiopancreatography. **(A,B)** Intraoperative findings of the fifth procedure in Case 2. The main pancreatic duct and branch pancreatic ducts were irregularly dilated (red arrow), and the proximal pancreatic duct was narrowed (black arrow); two pancreatic stents (5Fr-5 cm) (blue arrow) were placed for drainage after balloon dilatation of the stenosed area; **(C,D)** intraoperative image of the first ERP procedure in Case 6, she experienced spontaneous nipple intubation (the major papilla is marked with a blue arrow), which resulted in the penetration of the guide wire (black arrow) from the accessory nipple (red arrow), Incomplete PD confirmed by selective minor papilla intubation; **(E–G)** case 7 CT and pancreatography showed pancreatic duct dilatation (blue arrow), and the cyst (red arrow) communicate with the main pancreatic duct **(E,F)**, reexamination at 2 months after operation showed that the stent (black arrow) was in place and the cyst was absorbed **(G)**; **(H)** in Case 7, only the ventral pancreatic duct (red arrow) was visualized by the main papilla cannulation, and the accessory papilla cannulation showed the main pancreatic duct (blue arrow), but no communicating branches were found in the ventral and dorsal pancreatic ducts. Accordingly, the diagnosis of complete PD was made.

Experienced endoscopists (*n* = 3) performed all procedures. ERP was utilized to visualize pancreatic duct abnormalities using fluoroscopic imaging. Cannulation of the main pancreatic duct through the major duodenal papilla was the initial attempt, followed by cannulation of the minor papilla if the main duct was not visualized or only a short segment was identified (typically not extending beyond the midline of the abdomen). A dorsal pancreatic duct traversing the entire pancreas was indicative of pancreas divisum, further classified as complete (no communication between dorsal and ventral ducts, Figure 1H) or incomplete (presence of a short, thin communication channel, Figures 1C,D). Following definitive diagnosis, appropriate endoscopic interventions were performed, primarily involving the placement of pancreatic duct stents to facilitate unobstructed drainage of pancreatic secretions. In cases of PD with co-existing CP, procedures included minor papilla sphincterotomy or dilation alongside dorsal pancreatic duct stent placement.

### 2.3 Postoperative management

Following ERP, patients underwent continuous ECG monitoring for 8–24 h. Vital signs were closely observed, and patients were monitored for symptoms such as abdominal pain, vomiting, fever, bleeding, and abdominal distension. A routine 24 h postoperative fasting period was followed, with gradual dietary resumption contingent on normal amylase levels and the absence of abdominal pain. In cases of postoperative complications, the fasting period may be extended. Dynamic monitoring of complete blood count, liver and kidney function, and serum amylase levels was performed at 3 and 24 h postoperatively. Treatment was tailored based on the ERP procedure and any postoperative complications and could include antibiotics, hemostasis, pancreatic enzyme suppression, fluid replacement, and intravenous nutritional support.

### 2.4 Observation index

(1) Patient demographics and presenting symptoms, (2) ERP completion details including diagnostic findings, interventions performed, and treatment outcomes, (3) operative time measured from scope insertion to removal, and (4) post-procedural complications such as PEP, hyperamylasemia, cholecystitis, bleeding, and perforation.

### 2.5 Follow-Up of patients after procedure

Following the first ERP procedure for each child, the follow-up period commenced and continued until July 1, 2024, monitoring clinical symptoms, changes in imaging studies, the annual frequency of PEP episodes, and assess postoperative abdominal pain using a Visual Analogue Scale (VAS) ranging from 0 (no pain) to 10 (severe pain). VAS scores were further categorized as follows: 0–3 indicated mild pain with no sleep disturbance, 4–6 indicated moderate pain causing mild sleep disruption, and 7–10 indicated severe pain preventing sleep or causing nighttime awakenings due to pain.

### 2.6 Statistical analysis

Statistical analysis was performed using SPSS 25.0 software. Normally distributed continuous data were presented as mean ± standard deviation (SD) and were compared using *t*-tests. Conversely, non-normally distributed continuous data were presented as median with interquartile range (IQR) and were analyzed using Wilcoxon signed-rank tests. A *P*-value of less than 0.05 was considered statistically significant.

## 3 Results

### 3.1 General information

A total of 17 pediatric patients participated in the study (7 males, 10 females), with ages ranging from 4.4 to 15.9 years (mean: 10.0 ± 2.7 years). The lightest patient weighed 14 kg. All patients presented with upper abdominal pain. Additionally, 7 patients experienced vomiting, and 1 had a fever. Notably, no significant signs of exocrine or endocrine dysfunction (e.g., steatorrhea or diabetes) were observed. The duration of abdominal pain ranged from 6 to 48 months, with an average of 18.5 months. Importantly, none of the patients reported a history of unhealthy dietary habits or alcohol consumption.

Limited by high costs, genetic testing was performed in just one case. This identified a heterozygous mutation (c.194 + 2 T > C) in the *SPINK1* gene despite no familial history of pancreatitis reported by the parents. Notably, the patient's father had undergone prior pancreatic surgery, and the patient's sister also had CP but did not receive genetic testing.

Imaging with either computed tomography (CT) or MRCP identified irregular, narrow, or tortuous dilatation of the pancreatic ducts in all 17 patients. Pancreatic duct stones were present in twelve patients, while four exhibited morphological changes within the pancreas itself. Notably, only two patients displayed evident pancreatic calcifications on imaging. Additionally, three patients developed pseudocysts within the pancreas, with two of these cases demonstrating communication with the main pancreatic duct.

All 17 patients exhibited irregularities of the main pancreatic duct, manifesting as either narrowing, tortuosity, or dilatation. Two patients additionally presented with branch duct dilatation, and twelve had filling defects identified during imaging. Within this group, four patients were diagnosed with pancreatic divisum (three complete type and one incomplete type), and two had both pancreatic divisum and PBM (Table 1). MRCP successfully identified three cases of pancreatic divisum and both PBM cases; however, one case of pancreatic divisum was only detectable during ERP imaging and remained undiagnosed preoperatively by MRCP. ERP definitively confirmed all four diagnoses of pancreatic divisum.

**Table 1:** The clinical data and examination results of children with CP.

No.	Sex	Age (years)	disease duration (Months)	BMI (kg/m^2^)	Symptoms			Serum amylase (U/L)	Imaging		Imaging findings				Anatomical or genetic abnormalities		
					Abdominal pain	Emesis	Fever		MR	CT	Narrowing or dilatation of ducts	Pancreatic duct stones	Morphological pan-creatic alterations	Other	PD	PBM	Gene mutation
1	Male	13.7	18.0	13.6	+	−	−	1414.0	−	+	+	+	+	nil	−	−	−
2	Female	10.3	36.0	15.6	+	+	−	447.0	+	+	+	+	+	PCC	−	−	−
3	Male	10.2	6.0	15.9	+	−	−	580.0	+	−	+	+	−	nil	−	+	−
4	Male	10.9	12.0	17.3	+	−	−	232.0	+	−	+	+	+	nil	−	−	−
5	Male	10.5	48.0	17.8	+	+	−	272.0	+	−	+	−	−	nil	−	+	−
6	Female	4.4	18.0	13.2	+	+	−	1414.0	+	−	+	−	−	nil	+^a^	−	−
7	Female	7.8	24.0	13.7	+		−	91.0	+	−	+	−	−	nill	+	−	−
8	Female	14.5	48.0	24.2	+	+	−	1186.0	+	+	+	−	−	PCC	+^a^	−	−
9	Female	9.8	9.0	13.3	+	+	−	1055.0	+	+	+	+	−	PPC	−	−	+
10	Female	7.6	16.0	12.0	+	−	−	33.0	+	−	+		−	nil	−	−	−
11	Female	8.8	24.0	15.0	+	−	−	269.0	+	+	+	+	−	PPC	−	−	−
12	Male	15.9	14.0	26.2	+	−	−	124.0	+	+	+	+	−	PPC	−	−	−
13	Male	9.8	12.0	17.4	+	−	−	324.0	+	+	+		−	PPC	−	−	−
14	Male	9.6	18.0	14.1	+	−	−	124.0	+	+	+	+	−	nill	+^a^	−	−
15	Female	10.1	12.0	17.4	+	+	−	1931.0	+	+	+		−	nil	−	−	−
16	Female	8.4	36.0	14.2	+	+	−	108.0	+	−	+	+	−	nil	−	−	−
17	Female	7.8	16.0	13.22	+	−	−	105.0	+	−	+	+	−	nil	−	−	−

^a^ Complete pancreas divisum, PPC, pancreatic pseudocyst; PCC, pancreatic calcification.

### 3.2 Endoscopic therapy

Upon admission, the 17 pediatric patients underwent a comprehensive preoperative evaluation. Patients experiencing an acute attack phase received supportive measures, including nil per os (NPO), intravenous fluid resuscitation, and enzyme replacement therapy until their symptoms stabilized. Elective ERP was then scheduled. All 34 ERP procedures achieved successful cannulation (100% success rate). Four patients with pancreas divisum underwent minor papilla cannulation. For complete pancreas divisum with CP, the initial treatment involved minor papilla sphincterotomy and placement of a dorsal pancreatic duct stent. Incomplete pancreas divisum with CP was primarily managed with dual sphincterotomy (major and minor papillae) and placement of a minor pancreatic duct stent. Additional procedures, like balloon dilation of the major and minor papillae and pancreatic duct stenting, were performed as needed based on individual patient presentations.

ERP interventions included pancreatic duct stent placement in 34 patients, with dual stents placed in 5 cases. Pancreatic duct stone removal using stone retrieval balloons was performed in 24 patients, with analysis revealing the stones to be primarily composed of protein plugs. Additionally, endoscopic papillary balloon dilation (EPBD) was performed in 5 patients and endoscopic sphincterotomy (EST) in 11 patients (details in Table 2). All patients experienced short-term symptom relief following ERP treatment.

**Table 2:** The details of endoscopic procedures and follow-up results for patients with CP.

No.	Therapeutic details	Models of pancreatic duct stents	Post-ERP Complications	Post-operative follow-up			
				Are symptoms relieved?	Treatment interval (months)	Condition during treatment period	Follow-up time (months)
1	Stent placement + Stone extraction	5Fr-5 cm	None	Y	–	no symptoms	48
	Stent placement	7Fr-7 cm	None	Y	3	No symptoms, the stent dislodge spontaneously	
2	Stent placement + Stone extraction	5Fr-7 cm	None	Y	–	Pancreatitis-related abdominal pain 2 months after ERP	48
	Stent placement + Stone extraction	7Fr-6 cm	None	Y	4	Pancreatogenic ancreatitic pain 2 and 6 months after ERP	
	Stent placement + Stone extraction	7Fr-6 cm	None	Y	8	Pancreatitis-related abdominal pain 1 months after ERP	
	Stent placement + EST + Stone extraction	7Fr-5 cm	PEP	Y	5	No symptoms	
	Stent placement + Stone extraction	5Fr-5 cm, 5Fr-5 cm	None	Y	4	No symptoms	
3	Stent placement + EPBD + Stone extraction	5Fr-7 cm	None	Y	–	No symptoms, the stent dislodge spontaneously	30
	Stent placement + EST + Stone extraction	7Fr-5 cm	None	Y	6	No symptoms	
4	Stent placement + EST + Stone extraction	5Fr-5 cm	None	Y	–	No symptoms, the stent dislodge spontaneously	30
5	Stent placement + Stone extraction	7Fr-7 cm	None	Y	–	No symptoms	38
	Stent placement + EST + Stone extraction	5Fr-5 cm, 5Fr-7 cm	None	Y	12	No symptoms, the stent dislodge spontaneously	
	Stent placement + EST + Stone extraction	5Fr-7 cm, 7Fr-7 cm	None	Y	16	Pancreatitis-related abdominal pain 16 months after ERP, the stent dislodge spontaneously	
6	Stent placement + Stone extraction	5Fr-5 cm	Hyperamylasemia	Y	–	No symptoms	24
	Stent placement	5Fr-5 cm	None	Y	1	Pancreatitis-related abdominal pain 1 months after ERP, the stent dislodge spontaneously	
7	Stent placement	5Fr-5 cm	None	Y	–	No symptoms, the stent dislodge spontaneously	24
8	Stent placement	5Fr-7 cm	None	Y	–	No symptoms	16
	Stent placement + minor papillotomy	5Fr-5 cm	None		5	No symptoms, the stent dislodge spontaneously	
9	Stent placement + EPBD + Stone extraction	5Fr-7 cm	Hyperamylasemia	Y	–	No symptoms	28
10	Stent placement + EPBD + Stone extraction	7Fr-7 cm, 7Fr-7 cm	None	Y	–	No symptoms	18
11	Stent placement + EST + EPBD + Stone extraction	5Fr-7 cm	None	Y	–	No symptoms	26
12	Stent placement + EST + Stone extraction	5Fr-7 cm	None	Y	–	No symptoms, the stent dislodge spontaneously	
	Stent placement + EPSBD + Stone extraction	7Fr-5 cm	Hyperamylasemia	Y	8	No symptoms	24
	Stent placement + EPSBD + Stone extraction	7Fr-7 cm	PEP	Y	6	No symptoms	
	Stent placement + Stone extraction	7Fr-7 cm	None	Y	6	No symptoms	
	Stent placement + Stone extraction	7Fr-7 cm	None	Y	3	Pancreatitis-related abdominal pain 3 months after ERP	
13	Stent placement + EST + Stone extraction	5Fr-5 cm	None	Y	–	No symptoms	20
	Stent placement	7Fr-5 cm	None		3	Pancreatitis-related abdominal painn 1 months after ERP	
14	Stent placement + EST + EPBD + Stone extraction	7Fr-7 cm	Hyperamylasemia	Y	–	No symptoms	6
	Stent placement + Stone extraction	5Fr-5 cm, 7Fr-7 cm	None		1	Pancreatitis-related abdominal pain 1 months after ERP	
15	Stent placement + EPSBD	7Fr-5 cm	None	Y	–	No symptoms	6
16	Stent placement + EST	5Fr-7 cm	None	Y	–	No symptoms	16
17	Stent placement	5Fr-5 cm	Hyperamylasemia	Y	–	No symptoms	6
	Stent placement + EST	7Fr-5 cm	None	Y	3	Pancreatitis-related abdominal pain 3 months after ERP	

EST, endoscopic Sphincterotomy; EPBD, endoscoopic papillary balloon dilatation; EPSBD, endoscopic pancreatic sphincter balloon dilation; “-”For the first time, there is no treatment interval for a single endoscopic treatment or multiple endoscopic treatments.

### 3.3 Complications of ERP

Of the 17 pediatric patients, three developed PEP, and five exhibited post-procedural hyperamylasemia. Following the classification system established by Cotton et al. (11), all PEP cases were classified as mild and resolved swiftly with conservative interventions, including NPO, fluid resuscitation, and suppression of pancreatic enzyme secretion. Fortunately, no patients experienced complications like hemorrhage or perforation, and there were no mortalities associated with the procedures.

### 3.4 Follow-up data

All patients underwent immediate follow-up following their initial ERP procedure. The average follow-up duration was 24.0 months (range, 6–48 months), with three patients exceeding three years of follow-up. Seven patients experienced pancreatitis-related abdominal pain and required readmission during this period. The remaining ten patients exhibited well-controlled clinical symptoms, with no significant abdominal pain or acute pancreatitis episodes. At the time of publication, five patients had been followed for more than two years without disease recurrence and had discontinued treatment (They no longer required endoscopic interventions and were managed with dietary adjustments and regular outpatient follow-up).

Patients underwent an average of 2.2 stent replacements (range 1–5), with intervals between replacements lasting 3–12 months. Notably, imaging studies showed no significant worsening of pancreatic duct dilation during this period. One stent self-dislodged but was not replaced due to a lack of clinical symptoms or imaging progression at the three-month follow-up. Median VAS scores for pain significantly improved from 6 pre-procedure to 2 post-procedure (median difference: 4 points; *Z* = −3.580, *P* < 0.001). Annual pancreatitis episodes also significantly decreased from a mean of 2.3 to 0.5 after stent placement (*Z* = −3.628, *P* < 0.001). Among the 17 successfully treated patients, follow-up measurements of height, weight, and Body Mass Index (BMI) revealed a statistically significant increase in BMI, from a mean of 16.1 ± 3.7 kg/m² pre-treatment to 17.3 ± 3.7 kg/m² post-treatment (*t* = −8.933, *P* < 0.001) (Table 3).

Our findings demonstrate the efficacy of ERP with stent placement in managing pediatric pancreatic duct pathology. This approach resulted in symptom alleviation and improved clinical outcomes for the patients involved.

**Table 3:** Comparison of BMI and the number of pancreatitis attacks per year before and after treatment.

Variable	Before treatment	After	*P* value
Number of pancreatitis episodes per year, Median (IQR)	2.3 (1.0)	0.5 (0.7)	<0.001
BMI (kg/m^2^), (mean ± SD)	16.1 ± 3.7	17.3 ± 3.7	<0.001
Abdominal pain scores (VAS)	6 (2)	2 (2)	<0.001

Pre-treatment data were collected at the time of the first ERP and post-treatment data were collected at the last follow-up.

## 4 Discussion

CP, a debilitating disease characterized by irreversible pancreatic tissue damage and dysfunction of both endocrine (hormonal) and exocrine (digestive enzyme) functions, arises from various factors. Despite a low overall incidence in children (ranging from 1/200,000 to 1/500,000), with a prevalence of up to 29/500,000, both figures show a concerning upward trend in recent years (2, 5). Notably, unlike some adult presentations, research suggests no significant gender disparity in childhood CP (12). As a common justification for pediatric ERP (13–15), our institution, one of China's leading tertiary children's hospitals performing ERCP, has gained substantial experience diagnosing and treating this condition. We strongly believe ERP offers invaluable diagnostic capabilities. It provides high-resolution pancreatic and biliary imaging, which is crucial for pinpointing potential causes and defining anatomical details (16). Moreover, it possesses significant therapeutic potential, particularly in identifying PBM, pancreas divisum, and evaluating pancreatic duct strictures. Compared to traditional surgical procedures, ERP boasts advantages in effectiveness, minimal invasiveness, and safety, making it a more patient-acceptable option and a partial replacement for traditional interventions. Notably, several high-volume ERP centers have reported promising data, with success rates of duct cannulation in children under 1 year old reaching approximately 90% (14, 17, 18). However, this success rate decreases with lower body weight, suggesting an increased risk of cannulation failure in the major or minor papilla (18).

Common etiologies of CP in children encompass anatomical abnormalities, genetic predispositions, toxic/metabolic disorders, autoimmune conditions, and idiopathic factors (19). Notably, within the cohort of 17 pediatric patients who underwent endoscopic intervention in this study, none reported a history of alcohol consumption. However, congenital anatomical abnormalities and genetic defects were identified in 7 cases (41.2%), representing the primary etiologies.

This study demonstrates a potential therapeutic benefit of ERP with pancreatic duct stenting for managing CP-related abdominal pain in children. The majority of enrolled pediatric patients achieved clinical pain relief following the procedure. This finding aligns with established data, indicating that roughly 75% to 80% of CP patients experience abdominal pain during disease progression (20). Notably, one primary contributing factor to this pain is elevated pancreatic parenchymal pressure caused by pancreatic duct obstruction (21). Prior research suggests that approximately 70% of such patients can relieve symptoms through surgical or endoscopic pancreatic fluid drainage (22). It is important to acknowledge that the presentation of CP differs between children and adults. While adult patients with CP exhibit a higher incidence of steatorrhea (22.9%) (23) and diabetes (24), only a small percentage (approximately 1.3%) of pediatric CP patients develop diabetes, with a slower disease progression (3). This study did not observe any cases of steatorrhea or diabetes, and the included patients generally demonstrated normal growth and development. These observations may be attributed to the shorter disease course in children and their enhanced capacity for pancreatic tissue repair.

Pediatric CP diagnosis presents unique challenges. Pancreatic biopsy, while potentially informative, carries significant risks in children, and obtaining reliable pathological results can be difficult (25). Consequently, the diagnosis of pediatric CP relies heavily on a combination of clinical symptoms and imaging findings. The radiographic presentation of pediatric CP differs from that observed in adults. In this study, the most prevalent imaging abnormalities were tortuous pancreatic duct dilation and the presence of pancreatic duct stones, typically composed of protein cores. In contrast, pancreatic calcifications, more commonly seen in adult CP (26), were less frequent. Notably, abdominal CT and MRCP offer complementary diagnostic advantages. Abdominal CT excels at visualizing pancreatic calcifications and established pancreatic duct stones, while MRCP offers superior sensitivity for detecting pancreaticobiliary ductal variations and anatomical anomalies. While studies in adults have shown no statistically significant difference in the sensitivity and specificity of MRCP and abdominal CT for diagnosing CP (27), MRCP was the primary diagnostic modality in 16 of this study's cases, compared to only 1 case diagnosed by initial abdominal CT. Furthermore, MRCP identified three cases of pancreas divisum and two PBM, further highlighting its advantage over abdominal CT in pediatric CP diagnosis. This superiority likely stems from the fact that the primary etiologies of pediatric CP are often anatomical abnormalities such as pancreas divisum or PBM, which primarily manifest with pancreatic ductal changes—precisely the area where MRCP surpasses abdominal CT. Although ERP provides detailed visualization of the pancreatic ducts, branch ducts, and stones, advancements in MRCP technology have rendered it a diagnostic equivalent with a superior safety profile. Consequently, MRCP has largely supplanted ERP for the sole purpose of diagnosing CP (16). Therefore, MRCP should be the preferred imaging modality for evaluating suspected pediatric CP.

Endoscopic intervention is the mainstay of therapy for pediatric CP patients with pancreatic duct stones, strictures, or obstruction due to anatomical anomalies of the pancreaticobiliary system. However, management strategies in this population primarily rely on data extrapolated from adult studies, highlighting the need for further research and refinement of treatment protocols for pediatric CP (13). Consequently, the long-term efficacy of endoscopic interventions in pediatric CP requires ongoing validation. Our institution adheres to the Chinese CP Guidelines when performing endoscopic procedures on pediatric CP patients. Active endoscopic intervention is recommended for CP children with pancreatic duct stones, strictures, or pancreas divisum causing duct obstruction. For children experiencing recurrent episodes of CP (defined as greater than three occurrences per year) without duct stones or strictures, a diagnostic and potentially therapeutic endoscopic evaluation may be warranted following appropriate imaging studies (13). In cases of refractory pain associated with CP, ERP can be considered to assess pancreatic duct dilation and determine the feasibility of sphincterotomy of the major or minor papilla (1, 28). Successful decompression is primarily measured by pain control and a reduction in analgesic requirements. This study observed four patients who remained pain-free for over a year after treatment with spontaneous pancreatic duct stent expulsion. Reviewing their procedures revealed that only pancreatic duct stent placement and stone removal were performed without surgical incision or balloon dilation. This finding underscores the importance of maintaining pancreatic duct stent patency to ensure unobstructed pancreatic fluid drainage. Four additional patients experienced long-term pancreatic duct dilation resolved by stenting, with spontaneous stent expulsion and subsequent symptom alleviation. Due to the inherently narrow caliber of the pediatric pancreatic duct (typically less than 5 mm), forceful balloon dilation of strictures is contraindicated to minimize the risk of duct rupture. For particularly severe strictures impassable by standard pancreatic duct stents, biliary dilation catheters may be employed for initial dilation, followed by placement of pancreatic duct stents at the distal aspect of the stricture. These stents are typically replaced every 3–6 months until stricture resolution is achieved. In some cases, double plastic pancreatic duct stents may be a viable option (29) (Figures 1A,B).

Pancreatic pseudocysts (PPCs) are a well-recognized complication of acute and CP, post-surgical procedures, trauma, and tumors. These fluid collections, containing pancreatic juice or a high concentration of pancreatic enzymes, lack an epithelial lining on their cyst wall. Traditionally, surgical intervention has been considered the definitive treatment approach for PPCs. However, this strategy carries inherent invasiveness and is associated with complication rates ranging from 10% to 30% for procedures like gastric or jejunal anastomosis. Furthermore, mortality rates associated with surgical intervention for PPCs can range from 1% to 5% (30). Percutaneous drainage, performed under ultrasound or CT guidance, offers a less invasive alternative to surgical management. However, this approach necessitates external drainage and is not suitable for removing solid necrotic material within the pseudocyst. Studies report that approximately 53% to 62% of patients treated with percutaneous drainage eventually require surgical intervention due to complications such as bleeding, inadvertent puncture of neighboring organs, secondary infection, or the development of a pancreatic fistula (31).

ERP plays a valuable role in elucidating the status of the pancreatic duct and its potential communication with the pseudocyst. This information is crucial for selecting the most appropriate treatment modality. Studies estimate that approximately 80% of pancreatic pseudocysts demonstrate communication with the pancreatic duct (32). The findings of this study further support the safety and feasibility of ERP-guided management of pancreatic pseudocysts in conjunction with pediatric CP. Three cases of combined pancreatic pseudocyst and CP were identified in this study. Two of these cases exhibited significant communication with the main pancreatic duct, while the remaining case demonstrated no clear communication. All three patients underwent placement of a pancreatic duct stent via the major papilla. Following this intervention, all three patients experienced resolution of their pseudocysts and abdominal pain relief within a half-month period (Figures 1E–G).

The therapeutic efficacy of ERP with pancreatic duct stenting via the major papilla appears to depend on the presence of communication between the pancreatic pseudocyst and the main pancreatic duct. In cases where demonstrably patent communication exists, this minimally invasive approach offers a potentially curative solution by facilitating pseudocyst drainage and achieving favorable clinical outcomes. Furthermore, this study suggests that even without clear communication between the pseudocyst and the main pancreatic duct, ERP-mediated pancreatic duct stenting may be a viable treatment option for select patients. This approach may be particularly suitable for pseudocysts located in the pancreatic head with a diameter of less than 6 cm and no significant associated complications. The rationale behind this strategy lies in its ability to effectively reduce pancreatic fluid pressure within both the main and branch pancreatic ducts. This reduction in pressure helps prevent overflow of pancreatic fluid, ultimately achieving therapeutic goals (33).

This study identified four pediatric patients with CP coexisting with pancreas divisum. Following endoscopic intervention consisting of minor papilla sphincterotomy and placement of a dorsal pancreatic duct stent, all four patients experienced significant and sustained pain relief without any adverse events. These findings support the safety and feasibility of ERP-based treatment for CP in conjunction with PD. Pancreas divisum is a congenital anomaly characterized by an incomplete fusion of the pancreatic ducts during embryonic development. This can lead to a narrowed or malfunctioning minor papilla, potentially resulting in abdominal pain or even acute pancreatitis if the anomaly is not promptly addressed. In some cases, recurrent episodes of acute pancreatitis can progress to CP. Clinically, the presentation of CP combined with PD is often indistinguishable from isolated CP. Both conditions typically manifest with recurrent episodes of chronic abdominal pain, indigestion, and weight loss. The absence of distinct clinical features underscores the importance of diagnostic investigations to differentiate these entities.

Diagnosing CP in children is challenging early on because abdominal pain is usually mild to moderate and often not accompanied by elevated serum amylase levels. MRCP is currently an important non-invasive tool for diagnosing PD. However, a systematic review and meta-analysis published in 2014 found that the overall sensitivity of MRCP for diagnosing PD was only 52% (34). Despite being invasive, ERP remains the gold standard for diagnosing congenital biliary and pancreatic duct diseases like PD and PBM, as it provides clear imaging of the confluence of the biliary and pancreatic ducts and common channels (35, 36). Moreover, ERP allows for endoscopic treatment simultaneously.

Management of pancreas divisum co-existing with CP can be achieved through both endoscopic and surgical interventions. A meta-analysis (37) reported comparable overall remission rates, with 69.4% for endoscopic therapy and 74.9% for surgical intervention for symptomatic PD. However, the data did not reveal a significant difference between these approaches. Given the greater invasiveness and potential complications associated with surgery, endoscopic procedures are currently favored for the treatment of PD. This preference is further amplified in the pediatric population due to incomplete organ development and a lower tolerance for surgical trauma (38). Furthermore, studies and parental preferences often converge on ERP as the standard treatment modality for symptomatic PD in children. The cornerstone of successful therapy lies in achieving adequate pancreatic fluid drainage to alleviate pancreatic duct hypertension.

Prior studies have emphasized the importance of ensuring adequate pancreatic drainage to prevent complications, and sphincterotomy has been proposed as a method to achieve this goal (39). However, sphincterotomy is not without its limitations. This procedure carries inherent risks such as intraoperative bleeding, perforation, and retrograde cholangitis. Furthermore, sphincterotomy can potentially compromise sphincter and gallbladder function, leading to long-term complications like bile reflux and biliary pancreatitis (40). In this study, we posit that balloon or catheter dilation may be a more suitable approach for pediatric patients due to the inherently greater elasticity of the minor duodenal papilla in children than adults. Dilation offers the advantage of maximizing the structural preservation of the sphincter, thereby maintaining its physiological function and facilitating effective pancreatic fluid drainage.

Case 14 was the only instance in this study where minor papilla sphincterotomy became necessary due to complications encountered during stent replacement. This patient had a more protracted disease course (48 months) with a history of recurrent pancreatitis admissions. Imaging studies revealed findings consistent with chronic pancreatic inflammation. CP can lead to irreversible alterations in the pancreatic parenchyma and ducts, potentially resulting in functional impairment and decreased compliance of the minor duodenal papilla over time. In such cases, relying solely on dilation may be insufficiently effective and could even carry the risk of sphincter tear. Therefore, we propose that sphincterotomy may be a reasonable consideration for patients with a longer disease duration and evidence of sphincter dysfunction. Conversely, for children with a shorter disease course and no evidence of sphincter dysfunction, sphincterotomy should be avoided whenever possible, favoring a minimally invasive approach like balloon or catheter dilation.

ERP remains a valuable diagnostic and therapeutic tool; however, it is not without inherent risks. PEP represents the most common complication associated with ERP, with reported incidence rates in adult populations ranging from 3% to 15% (41). Other potential complications include infections like cholangitis and cholecystitis, bleeding, perforation, and risks associated with anesthesia (13, 42).

Our case series encouragingly demonstrated a lower incidence of PEP and elevated amylase levels than what is typically observed in adult populations. Furthermore, this study did not identify any statistically significant correlations between the occurrence of PEP and either patient age or the presence of structural abnormalities. Notably, other studies have reported potential associations between PEP and various intraductal procedural factors. These factors include difficulty cannulating the papilla, pre-emptive sphincterotomy of the papilla, sphincterotomy of the pancreatic duct, contrast injection into the pancreatic duct, and balloon dilation of the bile duct (43). These findings suggest that PEP may be more likely in procedures involving manipulations within the pancreatic ductal system.

Additional studies have suggested that CP may act as a protective factor against PEP, potentially due to a combination of pancreatic atrophy and decreased enzyme activity (44). A systematic review by Keane et al. (42) highlighted that for patients with CP, the risk of complications associated with ERP is minimized when the procedure is performed by endoscopists with expertise in “adult” procedures and at high-volume centers. Their study notably reported no ERCP-related complications in their patient population. At our institution, ERP procedures are conducted by pediatric gastroenterologists specializing in pediatric endoscopy, with a moderate annual case volume of approximately 50. Our experience has not yielded any reports of severe complications such as bleeding, perforation, or mortality. These findings collectively suggest that pediatric surgical endoscopists at tertiary pediatric hospitals, trained according to standardized protocols, can safely perform ERP in children.

CP necessitates a multidisciplinary approach to long-term management (42, 45). This study demonstrates that appropriately selected pediatric patients with CP who undergo ERP experience significant improvements in both height and weight following endoscopic intervention. Furthermore, the frequency of recurrent pancreatitis episodes is demonstrably reduced after ERP, leading to a noticeable improvement in quality of life. Following ERP in children, adherence to national guidelines for CP management remains essential. However, the development of a tailored follow-up protocol specifically for the pediatric population is warranted. Based on our clinical experience and the potential for pancreatic duct stent occlusion, we recommend stent replacement every 3–6 months. This initial stent replacement is typically followed by imaging reevaluation every 3 months to monitor for pancreatic duct dilation. If the child remains asymptomatic and imaging reveals no disease progression, clinical observation can be continued.

Endoscopic treatment may be suspended when spontaneous stent detachment occurs without subsequent episodes of significant pancreatic pain or when imaging reveals unobstructed pancreatic ducts with no concerning strictures, even if the stent remains in place and the patient experiences no pain during the treatment interval. In these scenarios, with potential obstruction resolved as evidenced by spontaneous stent expulsion or clear imaging, close clinical follow-up without further endoscopic intervention can minimize the treatment burden for the child in the absence of symptom recurrence.

## 5 Conclusion

The etiology of CP in children deviates from that observed in adults, with a primary emphasis on anatomical anomalies. Diagnosis currently hinges on clinical presentation and imaging studies, with MRCP emerging as the preferred modality for pediatric CP. As ERP technology has matured and its application in the pediatric population has expanded, endoscopic interventions such as sphincterotomy, pancreatic duct stenting, and stone removal have significantly improved both quality of life and body mass index BMI in appropriately selected children with CP. This study further underscores the safety, efficacy, and minimally invasive nature of ERP and related techniques as both diagnostic and therapeutic tools. However, to strengthen the generalizability of these findings, further clinical observation is warranted given the limitations imposed by the relatively small sample size and short follow-up duration of this study.

## Data Availability

The original contributions presented in the study are included in the article/Supplementary Material, further inquiries can be directed to the corresponding author/s.
